# A meta-analysis of client-therapist perspectives on the therapeutic alliance: Examining the moderating role of type of measurement and diagnosis

**DOI:** 10.1192/j.eurpsy.2020.67

**Published:** 2020-06-29

**Authors:** Libby Igra, Michal Lavidor, Dana Atzil-Slonim, Nitzan Arnon-Ribenfeld, Steven de Jong, Ilanit Hasson-Ohayon

**Affiliations:** 1 Department of Psychology, Bar-Ilan University, Ramat Gan, Israel; 2 Lentis Psychiatric Institute, Lentis Research & FACT noord- Groningen, The Netherlands

**Keywords:** Agreement, meta-analysis, therapeutic alliance

## Abstract

**Background:**

Clients and therapists often have different perspectives on their therapeutic alliance (TA), affecting the process and outcome of therapy. The aim of the present meta-analysis was to assess the mean differences between clients’ and therapists’ estimations of TA among clients with severe disturbances, while focusing on two potential moderators: client diagnosis and alliance instrument.

**Method:**

We conducted a systematic literature search of studies examining both client perspective and therapist perspective on TA in psychotherapy among people with schizophrenia spectrum disorders, personality disorders, and substance misuse disorders. We then analyzed the data using a random-effects meta-analytic model with Cohen’s *d* standardized mean effect size.

**Results:**

Heterogeneity analyses (*k* = 22, Cohen’s *d* = −.46, 95% confidence interval = .31–1.1) produced a significant *Q*-statistic (*Q* = 94.96) and indicated high heterogeneity, suggesting that moderator analyses were appropriate.

**Conclusions:**

Our findings show that the type of TA instrument moderates the agreement on TA between client and therapist, but there was no indication of the client’s diagnosis moderating the effect. The agreement between client and therapist estimations seems to be dependent on the instrument that is used to assess TA. Specific setting-related instruments seem to result in higher agreement between clients’ and therapists’ estimations than do more general instruments that are applied to assess TA.

## Introduction

The therapeutic alliance (TA) has been consistently shown to be one of the most important factors in psychotherapy, and a robust alliance-outcome link has been found across different therapy approaches [1]. Bordin’s [2] classic and pan-theoretical definition of the TA describes it as consisting of the following factors: the quality and strength of the affective bond between therapist and client, an agreement on therapy goals, and a consensus on how to attain those goals. Interpersonal conceptualizations view the therapeutic relationship as involving the ongoing negotiation of meanings between the therapist’s presence and the client’s subjective experience [3]. In this framework, the TA is a dyadic process in which clients and therapists mutually influence one another [3]. Although most studies to date have examined the client’s perspective on the TA [4], recently there has been growing interest in the therapist’s perspective as well [1], allowing for an investigation of their agreement/discrepancies**:** that is, the mean difference between client-therapist TA ratings [5–7].

The agreement between therapists’ and clients’ TA estimations plays an important role in determining both therapy process evaluations and therapy outcomes. Marmarosh and Kivlighan [8] found that agreement on the perceived TA at the beginning of the treatment predicted greater symptom change following treatment. By contrast, disagreements were related to less favorable perceptions of the therapeutic session from the side of the client. Similarly, Rubel et al. [7] found symptom distress following disagreements on TA estimations. The positive implications of agreement on TA between client and therapist have been shown and discussed both when TA is strong and when it is weak [9]. Highly similar estimations of TA were found to be related to desirable therapeutic outcomes such as improvement in interpersonal problems and decreases in symptomatic level [10]. Low similarity in estimations of or disagreement regarding the TA can imply the occurrence of therapeutic ruptures [11]. Recognizing these situations accurately might allow the therapist to take appropriate action to repair a therapeutic rupture and thus enhance the effectiveness of psychotherapy [12–14]. In exploring mean difference scores, it is important to note that a previous meta-analysis demonstrated that clients systematically rated their alliance higher than did their therapists (*d* = 0.63) [15]. The authors proposed that clients and therapists brought different perspectives to their evaluations, leading to similar but not identical estimations. For example, whereas therapists may have rated the TA with one client relative to their TA with other clients, clients may have compared the TA with the relationships they had with other health service providers. Although in recent years there has been growing interest in the TA from different perspectives, only one meta-analysis examination of client-therapist TA gaps was conducted, more than a decade ago [15]. The current meta-analysis aimed to extend previous findings and explore current developments of research in the field. Specifically, it is the first meta-analysis focusing on TA congruence in psychotherapy among clients with severe disturbances.

In line with previous meta-analyses on the topic, we defined client-therapist agreement as the degree to which client and therapist estimations converge or diverge, represented by client-therapist mean difference scores of the TA [15]. The current meta-analysis considered both psychiatric diagnosis and type of scale as possible moderators in the association of the congruence between clients` and therapists` TA estimations. Interestingly, in the previous meta-analysis on client-therapist agreement, it was found that clients with substance abuse problems tended to have larger rating discrepancies with their therapists than did clients who had severe disturbances as classified by hospitalization setting or specific diagnosis (e.g., schizophrenia, borderline personality disorder, bipolar disorder, eating disorders, brain injuries, and severe mental disturbance) [15]. The authors of this meta-analysis suggested that differences in setting and costs of therapy, related to the different disorders, may have explained the results. That is, clients with disorders that were considered mild and who might have received therapy in university settings at low or no cost, and clients with substance abuse problems who were generally provided treatment free of charge, might have rated the TA highly out of gratitude or out of fear of offending their therapists [15]. Although the authors considered the possible differences in setting and costs of therapy, the impact of the specific diagnosis remained unclear.

The current work aimed to deepen the understanding of the role of specific diagnosis on the agreement on TA. However, whereas Tryon, Blackwell, and Hammel [15] categorized disturbances into three levels of severity (mild, moderate, and severe) according to setting and diagnosis, we sought to extend existing understandings of agreement on TA in psychotherapy by considering three diagnostic groups: clients diagnosed with schizophrenia spectrum disorders, personality disorders, and substance misuse disorders. All three are considered serious disorders that cause pervasive disruptions in social skills and functioning [16] and therefore pose special challenges to TA. Thus, among the many challenges faced by individuals with schizophrenia, personality disorders, and substance misuse disorders, of particular interest for the purposes of the current study were the documented interpersonal ones [17–18]. Of note, it may be that each diagnostic group represents a specific cluster of symptoms that have a different effect on agreement between client and therapist. For example, in schizophrenia, lack of agreement between client and therapist with regard to TA can be attributed to mismatch in narratives regarding the client and therapist roles [19]; in personality disorders, the activation of maladaptive interpersonal patterns may greatly affect the TA [20–22]; and in substance misuse disorders, special issues of trust and motivation may exist [23].

In addition to the examination of diagnosis as a possible moderator, different scales conceptualize TA differently and emphasize slightly different aspects of the therapeutic relationship [1], suggesting that type of TA measurement is a moderator. It should be noted that there are more than 30 different instruments that assess TA [9], and even when evaluating the most commonly used instruments, their shared variance has been shown to be less than 50% [24]. The different instruments that are used to assess TA may differ both in their underlying rationale and in their psychometric properties. The most commonly used scale is the Working Alliance Inventory (WAI)[25], which relies on Bordin’s [2] definition both theoretically and operationally and contains an identical number of items for each alliance subscale (Task, Goals, and Bond). Other scales are based on various theoretical conceptualizations and may emphasize slightly different aspects of the relationship in accordance with the purpose and method that led to their development. For example, the scale that assesses the therapeutic relationship in community mental health care (Scale to Assess the Therapeutic Relationship [STAR]) [26] was developed in an attempt to take into account specific aspects of the relationship in psychiatric settings, such as greater heterogeneity of treatment components and goals, increased variability of setting, and the institutional responsibility of the clinician [26]. In addition to the possible differences in content between the scales, each scale has slightly different items for clients` and therapists` versions. For example, the California Psychotherapy Alliance Scale (CALPAS; [27]) has four subscales (Patient Working Capacity, Patient Commitment, Working Strategy Consensus, and Therapist Understanding and Involvement) that are assessed in both client and therapist versions, using different wording and phrases for the items in each version. Given these considerations and the complexity of measuring TA [1], it might be beneficial to explore how the different scales influence the congruence between clients` and therapists’ estimations of TA.

The present meta-analysis explored agreement on TA between clients and their therapists among individuals with serious mental illness, focusing on two potential moderators: client diagnosis and the type of instrument that assesses TA.

## Methods

### Literature search

A systematic literature search was performed in accordance with Preferred Reporting Items for Systematic Reviews and Meta-analyses (PRISMA) guidelines [28]. The literature search was conducted on the PsycINFO, PubMed, and Google Scholar databases for studies appearing between January 2006 and December 2018. The current meta-analysis continued and extended the previous examination of client-therapist congruence [15]; as such, we included studies published after the time of that publication. The key words were (a) "working alliance,” “therapeutic alliance,” or “therapeutic relationship”; (b) “patient” or “client”; and (c) “therapist” or “counselor.”

### Study selection: inclusion and exclusion criteria

Our inclusion criteria were as follows: (a) ratings of the TA from both client perspective and therapist perspective had to be reported, (b) studies had to include at least eight individual sessions of psychotherapy, (c) studies had to have a group design of at least 10 participants, (d) patients had to be 18 years of age or older, and (e) studies had to be published in English, (f) over 60% of the subjects had to be diagnosed with schizophrenia spectrum disorders, personality disorders, or substance misuse disorders according to *Diagnostic and Statistical Manual of Mental Disorders* (4th ed., text rev.) criteria. We excluded studies that used alliance measures that were developed for the purpose of a specific study and were not used elsewhere. When more than one assessment of TA was made, we took the first assessment.

**Figure 1.**
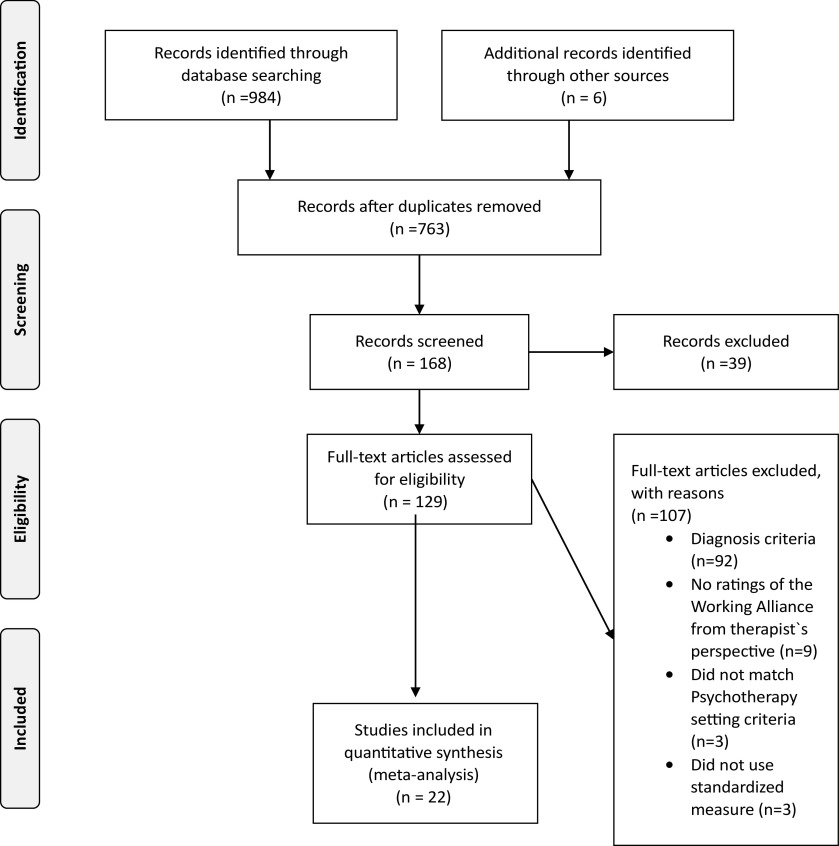
PRISMA 2009 flow diagram.

The initial search yielded 763 articles. Two independent coders conducted the initial screening by examining titles and removing records that clearly did not match the inclusion criteria. Next, each coder examined the abstracts and made a decision as to whether they met the inclusion criteria. In cases of disagreement, or in cases where the abstract did not include enough information to allow agreement, the full text was obtained. This process enabled the identification of 22 studies, *n* = 3,647. Figure 1 presents the PRISMA chart of study selection.

### Sample-level information

We included sample information regarding study year, authors, diagnosis, sample size, alliance measures, effect size, and time of measurement when available, as can be seen in Table 1.

**Table 1.** Studies included in meta-analysis.

Study	*N*	Diagnosis	TA scale	Time	Cohen’s *d*
Wittorf et al. [29]	160	Schizophrenia spectrum	BSQ	Third session	0.73
Berry et al. [30]	104	Psychosis and cannabis dependence or abuse	WAI-S	After 1 month	0.27
Wittorf et al. [31]	134	Schizophrenia spectrum	BSQ	Third session	0.8
Cavelti et al. [32]	266	Schizophrenia spectrum	STAR		0.08
Jung et al. [33]	96	Schizophrenia spectrum	HAQ	Fifth session	0.55
Evans-Jones et al. [34]	48	Schizophrenia spectrum	WAI	Sessions 2–9	0.75
Johansen et al. [35]	84	Schizophrenia spectrum	WAI-S		−0.08
Barrowclough et al. [36]	232	Schizophrenia spectrum	WAI-S	Fourth session	0.31
Lysakeret al. [37]	80	Schizophrenia spectrum	WAI-S	Selected session from first month	0.54
Ruchlewska et al. [38]	290	Schizophrenia spectrum	WAI		0.27
Mulligan et al. [39]	42	Schizophrenia spectrum	WAI-S		0.84
Dunn et al. [40]	60	Schizophrenia spectrum	CALPAS	Third session	0.89
Cook et al. [41]	346	Alcohol misuse	WAI	Third session	0.21
Nissen-Lie et al. [42]	379	Personality disorder	WAI-S	First session	0.18
Tufekcioglu et al. [43]	168	Personality disorder	WAI-S	Third session	0.5
Bedics et al. [44]	202	Borderline personality	CALPAS	First session	0.94
Huddy et al. [45]	98	Schizophrenia spectrum	WAI-S	First session	0.26
Theodoridou et al. [46]	232	Personality disorder	STAR	Fourth session	0–0.02
Spinhoven et al. [47]	156	Borderline personality	WAI		0
Lysaker et al. [48]	80	Schizophrenia spectrum	WAI-S		0.41
Richardson-Vejlgaard et al. [49]	70	Borderline personality	WAI-S	After 3 months	0.76
Artkoski & Saarnio [50]	320	Substance misuse	SQ	After 1 month	0.95

Abbreviations: BSQ, Bern Session Questionnaires; CALPAS: California Psychotherapy Alliance Scale; HAQ, Helping Alliance Questionnaire; SQ: Session Questionnaire; STAR, Scale to Assess the Therapeutic Relationship; WAI, Working Alliance Inventory.

Abbreviations: BSQ, Bern Session Questionnaires; CALPAS: California Psychotherapy Alliance Scale; HAQ, Helping Alliance Questionnaire; SQ: Session Questionnaire; STAR, Scale to Assess the Therapeutic Relationship; WAI, Working Alliance Inventory.

### Effect size

We identified 22 records (*n* = 3,647). We analyzed the data using a random-effects meta-analytic model with Cohen’s *d* standardized mean effect size. Further, we conducted moderator analyses with client diagnosis and alliance instrument as potential categorical moderators of the total effect size.

## Results

### Study selection and characteristics

Figure 1 displays article identification and inclusion, and Table 1 includes detailed study characteristics at the individual study level. Twenty-two studies met inclusion criteria for this meta-analysis, for a total of *n* = 3,647 participants.

### Sensitivity analyses and publication bias

A visual examination of the funnel plot and forest plot revealed heterogeneous effect sizes. All studies were retained for analyses. Trim-and-fill analyses indicated no change in the effect size after looking for extreme values, suggesting that results were robust against publication bias.

### Main analyses

**Figure 2.**
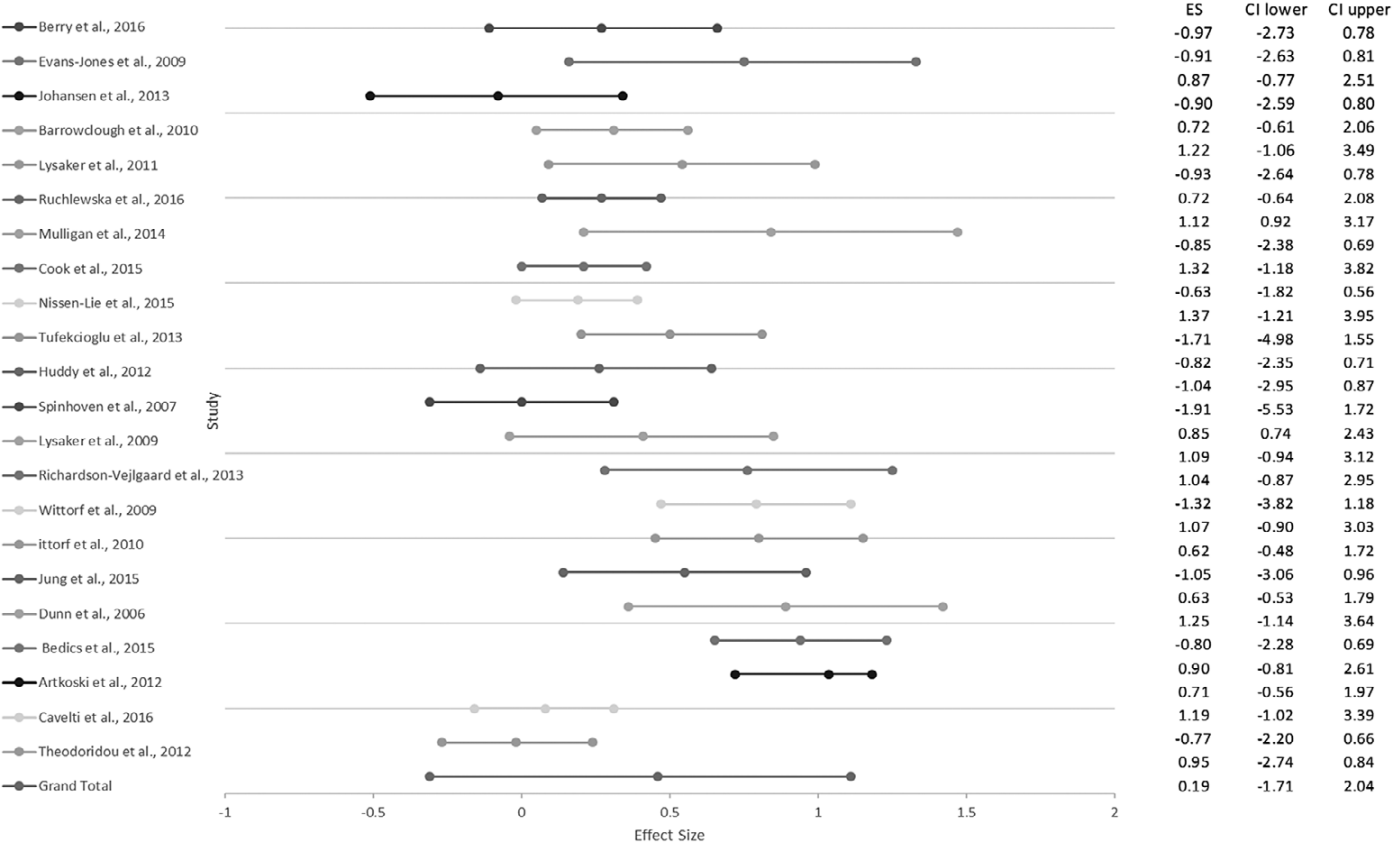
Forest plot of studies included in the meta-analysis.

Results indicated a medium effect size for the mean client-therapist difference in their TA estimations (*k* = 22, Cohen’s *d* = −.46, 95% confidence interval [CI] = .31–1.1). Heterogeneity analyses produced a significant *Q*-statistic (*Q* = 94.96) and a high amount of heterogeneity, as indicated by the *I* ^**2**^ statistic (*I* ^**2**^ = 77.88%), suggesting that moderator analyses were appropriate. Figure 2 presents the forest plot of meta-analytic results.

### Moderator analyses

We assumed that the diagnosis could be a moderator that would subdivide the studies’ effect sizes. Fourteen studies included samples with schizophrenia spectrum disorders, five studies included samples with personality disorders, and three studies included samples with substance misuse disorders. Heterogeneity analyses for the three subgroups produced significant *Q* statistics and a medium to high amount of heterogeneity. The results were *Q* = 31.14, *I* ^**2**^ = 58.25% for the schizophrenia spectrum disorders subgroup; *Q* = 26.54, *I* ^**2**^ = 84.92% for the personality disorders subgroup; and *Q* = 36.22, *I* ^**2**^ = 94.47% for the substance misuse disorders subgroup. These results indicate that diagnosis was not a moderator of the client-therapist mean difference.

Next, we assumed that the measure type could be a moderator that would subdivide the studies’ effect sizes. Fourteen studies used the WAI [25]; two studies used the STAR in community mental health care [26]; and six studies used other measures, that is, the CALPAS [27], the Helping Alliance Questionnaire (HAQ) [51], the Bern Session Questionnaire (BSQ) [52], and the Session Questionnaire (SQ) [53]. Heterogeneity analyses for the three subgroups produced nonsignificant *Q* values, indicating three different homogeneous effect sizes. The results were *Q* = 20.5, *I* ^**2**^ = 0%, *d* = .37, and 95% CI = .21–.78 for studies using the WAI, indicating a medium effect size for the client-therapist mean difference. For studies using the STAR, the results were *Q* = 19.87, *I* ^**2**^ = 0%, *d* = .03, and 95% CI = .23–.29, indicating that there was no effect size for the client-therapist mean difference. And for studies using the other measures (CALPAS, HAQ, BSQ, and SQ), results were *Q* = 3.7, *I* ^**2**^ = 0%, *d* = .81, and 95% CI = 0.44–1.26, indicating a large effect size for the client-therapist mean difference.

## Discussion

The present meta-analysis included a total of 22 studies assessing TA by both therapists and clients diagnosed with schizophrenia spectrum disorders, personality disorders, and substance misuse disorders. Consistent with previous investigations of TA among individuals with other mental disorders [15], we found that the clients in the current meta-analysis across the three diagnostic groups tended to estimate TA as somewhat higher than did their therapists. With regard to the possible moderating roles of client diagnosis and type of TA measurement in TA agreement, the findings supported the moderating role of type of measurement, but not of client diagnosis.

The finding that clients tend to estimate their alliance as somewhat higher than do therapists may represent a general psychotherapy bias, evident in both within [54] and between [15] designs. This bias can be attributed to the setting and different frameworks of each participant in the relationship, which stem from different expectations and different groups of comparisons [15]. On the therapist side, this gap has been suggested to represent a “better safe than sorry” attitude [54], according to which exercising caution enables therapists to be sensitive and hopefully more accurate regarding their clients’ fluctuations. From the client perspective, it may be that their expectations are based on previous experiences with stigmatizing health providers, especially among people with schizophrenia [55]. As such, against this kind of expectation, a positive experience in psychotherapy may be overly highly evaluated.

Our findings also suggest that the TA measurement type moderates the agreement between client-therapist estimations of the TA. The scale that assesses TA in community mental health care, that is, the STAR [26], showed no effect size for the differences between client and therapist perspectives on TA, implying no discrepancies between them. The STAR was specifically developed to assess the relationship between a variety of types of clinicians, and patients with severe mental illness, in community care settings. It was developed on the basis of a study comprising qualitative semi-structured interviews regarding TA, among therapists and clients with serious mental illness. The STAR includes 12 items consisting of three factors: positive collaboration and chemistry, in both client and therapist versions of the scale; positive clinical input, in the client version; and emotional difficulties, in the therapist version. Of note, the STAR was specifically developed to assess the relationship between a variety of professionals and patients with severe mental illness, in community care settings. Reflecting on the conceptual emphasis of this scale, it seems to emphasize slightly different aspects of TA that are critical for psychotherapy with individuals who have severe mental illness, for example, the perceived openness, trust, and honesty of the relationship from both sides (i.e., “My clinician and I share an honest relationship” and “My patient and I share a trusting relationship”). These factors are assumed to be more challenging for relationships with individuals who have severe disturbances, yet they were found to be crucial for creating shared meaning and enhancing TA [19,34]. In addition, the STAR includes a focus on understanding the meaning of the client’s subjective experience (“I believe my clinician has an understanding of what my experiences have meant to me”). This emphasis on the importance of making sense of the subjective experience is in line with recent research on people with schizophrenia showing that metacognitive mutual exploration of meaning enhanced TA and outcomes [56].

The WAI [25] produced a weak to medium effect size, implying that clients rated the alliance as somewhat higher than did their therapists. The four other scales (CALPAS, BSQ, HAQ, and SQ) included in our analysis yielded a large effect size implying larger discrepancies between clients and therapists when using these scales; it could be that their psychometric and theoretical orientations led to less agreement between clients and therapists. Reflecting on the differences between scales, it seems that in comparison to the STAR, the WAI and the other four scales mentioned above refer to more general aspects of the TA which are beyond the specific characteristic of psychiatric settings that provide services to people with serious mental illnesses. For example, the CALPAS scale includes items that focus on the general idea of treatment (i.e., “How much did you find yourself thinking that therapy was not the best way to get help with your problems?” or “Did the treatment you received in this session match with your ideas about what helps people in therapy?”).

In sum, it is possible that the reason the STAR (as opposed to the other measurements) yielded no gap between client and therapist estimations was its sensitivity to specific aspects of the TA with individuals who have serious mental illnesses in psychiatric settings. Instruments that are specific and sensitive to population and setting seem to be most appropriate when evaluating dyadic constructs among individuals with severe disturbances. Specifically, it seems that focusing on aspects of openness, trust, and honesty of the therapeutic relationship, and focusing on understanding the subjective meaning of the experiences, contribute to better agreement.

The finding that diagnosis did not moderate the agreement between client and therapist estimations of the TA may imply a trans-diagnostic phenomenon. A previous meta-analysis found that the severity of the disturbance as classified mainly according to setting groups moderated TA congruence, but specific diagnosis groups were not assessed [15]. In line with our findings, Atzil-Slonim et al. [54] found that preexisting symptomatology or personality disorder diagnoses did not moderate client-therapist agreement on TA. One possible explanation is that agreement is partially based on reflective abilities, an area in which clients with serious mental disorders often face challenges [18,57]. The clients included in the current meta-analysis might have shared these difficulties in reflecting on self and other, in a way that may have affected agreement, beyond specific diagnosis. Alternatively, it could be that therapists react similarly to clients with different diagnoses, assuming that they share similar difficulties in interpersonal relationships. However, the possible moderating effect of diagnosis cannot be fully ruled out as the current examination was limited to a relatively small number of diagnoses.

### Limitations and future directions

When considering the findings of the current meta-analysis, a few limitations should be taken into account. First, we used only one timepoint of assessment of TA (mostly at the beginning of therapy). Recent investigations have highlighted the importance of longitudinally tracking the TA in order to capture the complex dynamics of the dyadic perspective over the course of treatment [7,54,58]. Second, comparisons were made on a group level, without considering each dyadic mean score. Looking at the dyadic level (e.g., a small gap can reflect high estimations from both sides or low estimations from both sides) may have shed further light on these differences. In addition, it should be noted that therapeutic setting and diagnosis may be confounders, as some psychiatric settings aim to provide treatment for specific populations (e.g., substance misuse clinics). It is possible that diagnosis-related findings were obscured due to the effects of psychiatric setting, and data regarding the setting were not always available in the studies and, as such, were not entered into the current analysis. Finally, there are additional moderators that could potentially affect agreement between clients and therapists, such as process variables (e.g., therapy orientation and length of therapy), client variables (e.g., symptoms and personality characteristics), and therapist variables (e.g., experience and personal characteristics), which were not tested in the current meta-analysis.

### Summary and implications

With these limitations in mind, our meta-analysis suggests that clients with serious mental illness tend to have higher estimations of TA than do therapists, a finding that is also often found among clients without such psychiatric disorders. The level of agreement between client and therapist estimations seems to be dependent on the instrument used to assess TA, with no indication of client’s diagnosis moderating effect.

When considering agreement on TA, it is important to take into consideration the different aspects of TA that are emphasized in the different TA measurements. In terms of using the TA rating as a means of feedback, it could be beneficial for therapists to take into consideration specific items and concepts of TA when interpreting the meaning of the gaps between them and their clients. Specifically, when relating to more general aspects of TA, therapists can expect to see larger gaps between them than when relating to more context-specific aspects such as trust, openness, and meaning-making of subjective experiences.
